# Supplementary figures and images for: Mortality and loss to follow-up among Tuberculosis patients on treatment in Meru County, Kenya: a retrospective cohort study
Source: PLOS Glob Public Health. 2025 Mar 10;5(3):e0003896. doi: 10.1371/journal.pgph.0003896 (PMC11892847; doi:10.1371/journal.pgph.0003896)

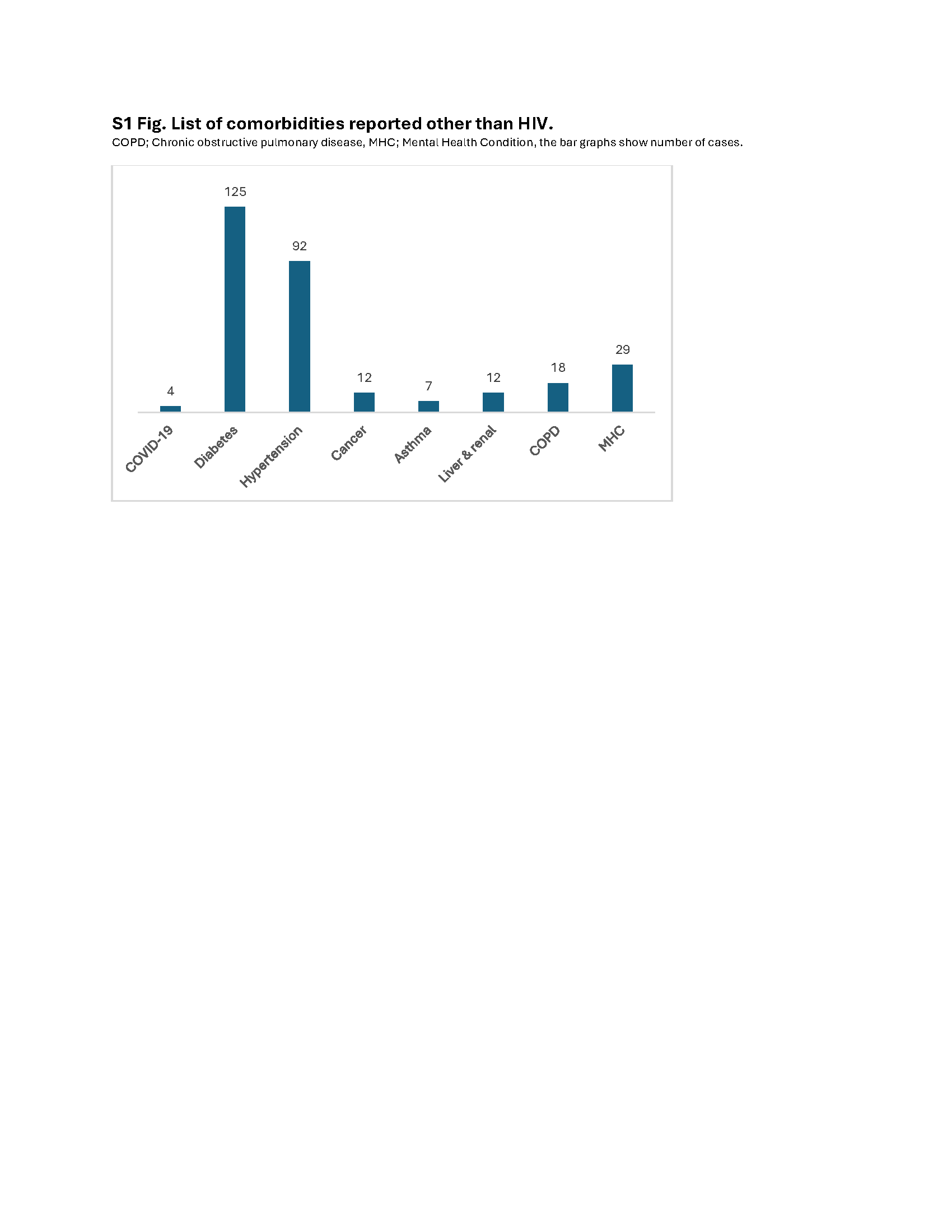

Supplement: S1 Fig — (TIFF) [file pgph.0003896.s001.tiff]

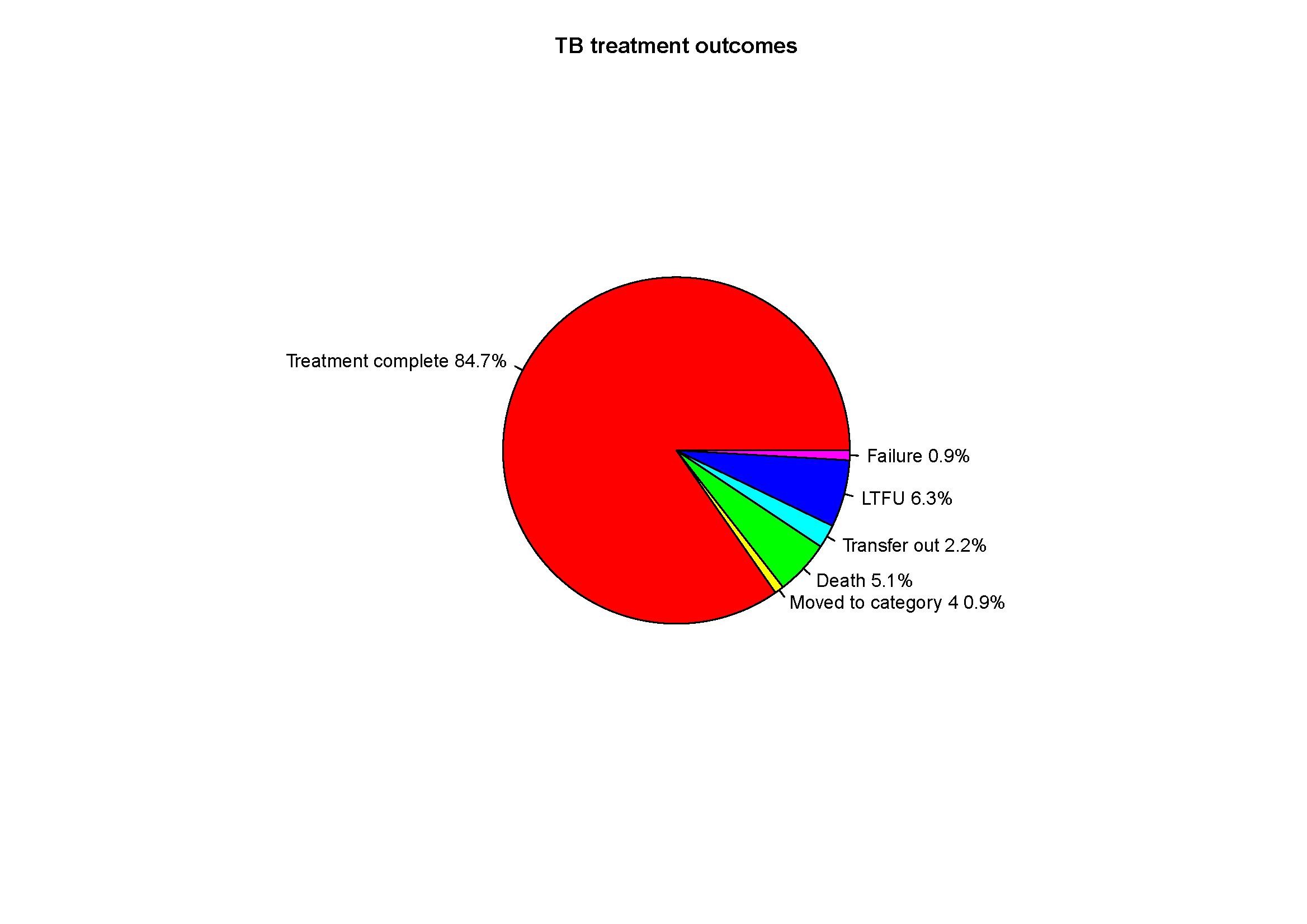

Supplement: S2 Fig — (TIFF) [file pgph.0003896.s002.tiff]
