# Supplementary material for: Mortality and loss to follow-up among Tuberculosis patients on treatment in Meru County, Kenya: a retrospective cohort study
Source: PLOS Glob Public Health. 2025 Mar 10;5(3):e0003896. doi: 10.1371/journal.pgph.0003896 (PMC11892847; doi:10.1371/journal.pgph.0003896)
Supplement: S1 Table — (DOCX) [file pgph.0003896.s003.docx]

**S1 Table. Participants characteristics stratified by age group.**

| **Characteristics** | **Age in years** | | | | | |  |
| --- | --- | --- | --- | --- | --- | --- | --- |
|  | **15 to 24 (9233)** | **25 to 34 (11830)** | **35 to 44 (8619)** | **45 to 54 (4383)** | **55 to 64 (2175)** | **≥65 (1780)** | **P-value** |
| Sex |  |  |  |  |  |  |  |
| Male | 6169 (22) | 8715 (32) | 6499 (24) | 3375 (12) | 1614 (5.9) | 1236 (4.5) | <0.001 |
| Female | 3064 (29) | 3115 (30) | 2120 (20) | 1008 (9.7) | 561 (5.4) | 544 (5.2) |  |
| Year of starting TB treatment |  |  |  |  |  |  |  |
| 2012 | 730 (26) | 1026 (36) | 562 (20) | 302 (11) | 145 (5.1) | 95 (3.3) | <0.001 |
| 2013 | 822 (26) | 1045 (33) | 673 (21) | 308 (9.8) | 164 (5.2) | 125 (3.9) |  |
| 2014 | 876 (26) | 1097 (33) | 714 (21) | 349 (10) | 163 (4.9) | 138 (4.1) |  |
| 2015 | 800 (26) | 1006 (33) | 628 (21) | 322 (11) | 145 (4.8) | 124 (4.1) |  |
| 2016 | 799 (27) | 977 (33) | 604 (21) | 280 (9.5) | 161 (5.5) | 118 (4.0) |  |
| 2017 | 1008 (26) | 1174 (31) | 846 (22) | 406 (11) | 215 (5.6) | 177 (4.6) |  |
| 2018 | 1009 (24) | 1297 (31) | 931 (22) | 516 (12) | 244 (5.7) | 253 (5.9) |  |
| 2019 | 934 (24) | 1155 (30) | 938 (24) | 457 (12) | 221 (5.7) | 174 (4.5) |  |
| 2020 | 717 (21) | 1034 (31) | 850 (25) | 403 (12) | 209 (6.2) | 169 (5.0) |  |
| 2021 | 726 (20) | 1012 (28) | 896 (25) | 513 (14) | 237 (6.6) | 193 (5.4) |  |
| 2022 | 812 (21) | 1007 (26) | 977 (26) | 527 (14) | 271 (7.1) | 214 (5.6) |  |
| Treatment facility type |  |  |  |  |  |  |  |
| Public health facility | 7430 (25) | 9466 (32) | 6711 (22) | 3371 (11) | 1627 (5.4) | 1367 (4.6) |  |
| Private health facility | 1674 (23) | 2043 (28) | 1714 (24) | 928 (13) | 509 (7.0) | 394 (5.4) | <0.001 |
| Prisons | 129 (16) | 321 (41) | 194 (25) | 84 (11) | 39 (4.9) | 19 (2.4) |  |
| BMI group |  |  |  |  |  |  |  |
| Undernourished (BMI<18.5) | 5466 (26) | 6671 (31) | 4747 (22) | 2413 (11) | 1134 (5.3) | 900 (4.2) | <0.001 |
| Normal (BMI 18.5 to 24.9) | 2853 (23) | 4001 (32) | 2941 (23) | 1440 (11) | 736 (5.9) | 602 (4.8) |  |
| Overweight (BMI ≥25) | 219 (14) | 390 (26) | 420 (27) | 248 (16) | 151 (9.9) | 101 (6.6) |  |
| Unknown/missing | 695 (27) | 768 (30) | 511 (20) | 282 (11) | 154 (6.0) | 177 (6.8) |  |
| TB diagnosis |  |  |  |  |  |  |  |
| Bacteriologically confirmed | 7026 (26) | 8823 (33) | 6036 (23) | 2723 (10) | 1191 (4.5) | 800 (3.0) | <0.001 |
| Clinical signs and X-ray | 2207 (19) | 3007 (26) | 2583 (23) | 1660 (15) | 984 (8.6) | 980 (8.6) |  |
| Patient category |  |  |  |  |  |  |  |
| New case | 8502 (25) | 10506 (31) | 7521 (22) | 3870 (11) | 1938 (5.7) | 1642 (4.8) | <0.001 |
| Re-treatment after relapse | 281 (12) | 711 (30) | 743 (32) | 351 (15) | 158 (6.8) | 89 (3.8) |  |
| Re-treatment after LTFU | 211 (25) | 324 (38) | 187 (22) | 66 (7.8) | 47 (5.5) | 16 (1.9) |  |
| Transfer in | 194 (30) | 225 (35) | 113 (17) | 69 (11) | 25 (3.8) | 26 (3.9) |  |
| Treatment after failure | 45 (22) | 64 (31) | 55 (27) | 27 (13) | 7 (3.4) | 7 (3.4) |  |
| Type of TB |  |  |  |  |  |  |  |
| Pulmonary TB | 8237 (25) | 10591 (32) | 7602 (23) | 3762 (11) | 1801 (5.4) | 1406 (4.2) | <0.001 |
| Extra-Pulmonary TB | 996 (22) | 1239 (27) | 1017 (22) | 621 (13) | 374 (8.1) | 374 (8.1) |  |
| HIV status |  |  |  |  |  |  |  |
| Negative | 8192 (27) | 9415 (31) | 6186 (20) | 3112 (10) | 1692 (5.6) | 1589 (5.3) | <0.001 |
| Infected | 766 (11) | 2108 (31) | 2198 (32) | 1149 (17) | 430 (6.4) | 122 (1.8) |  |
| Unknown/missing | 275 (26) | 307 (29) | 235 (22) | 122 (12) | 53 (5.0) | 69 (6.5) |  |
| Other comorbidity |  |  |  |  |  |  |  |
| None | 9215 (24) | 11796 (31) | 8549 (23) | 4322 (11) | 2136 (5.7) | 1722 (4.6) | <0.001 |
| Yes | 18 (6.4) | 34 (12) | 70 (25) | 61 (22) | 39 (14) | 58 (21) |  |
| On recreation drugs |  |  |  |  |  |  |  |
| No | 9100 (25) | 11393 (31) | 8050 (22) | 4112 (11) | 2068 (5.7) | 1703 (4.7) | <0.001 |
| Yes | 133 (8.3) | 437 (27) | 569 (36) | 271 (17) | 107 (6.7) | 77 (4.8) |  |
| Direct observed treatment (dot) |  |  |  |  |  |  |  |
| Family-based | 8745 (25) | 10986 (31) | 7945 (22) | 4064 (11) | 2022 (5.7) | 1653 (4.7) | <0.001 |
| Community health Volunteer | 68 (26) | 87 (33) | 54 (21) | 27 (10) | 11 (4.2) | 15 (5.7) |  |
| Healthcare worker | 420 (18) | 757 (32) | 620 (26) | 292 (12) | 142 (6.1) | 112 (4.8) |  |
| Treatment regimen |  |  |  |  |  |  |  |
| 2RHZE/4RH | 8838 (25) | 11172 (31) | 8120 (23) | 4134 (11) | 2048 (5.7) | 1699 (4.7) | <0.001 |
| 2SRHZE/1RHZE/5RHE | 259 (19) | 487 (36) | 336 (25) | 147 (11) | 81 (6.0) | 40 (2.9) |  |
| 2RHZ/4RH | 71 (23) | 91 (29) | 62 (20) | 50 (16) | 18 (5.9) | 12 (3.9) |  |
| RHZE/10RH | 45 (23) | 39 (20) | 52 (27) | 28 (14) | 16 (8.2) | 15 (7.7) |  |
| Others | 20 (13) | 41 (26) | 49 (31) | 24 (15) | 12 (7.5) | 14 (8.8) |  |
| Nutritional support |  |  |  |  |  |  |  |
| No food support | 1900 (26) | 2464 (34) | 1508 (21) | 736 (10) | 373 (5.1) | 287 (3.9) | <0.001 |
| Therapeutic/Supplementary food | 4245 (28) | 4675 (31) | 3114 (21) | 1502 (10) | 764 (5.1) | 624 (4.2) |  |
| Counselling only | 3088 (20) | 4691 (30) | 3997 (25) | 2145 (14) | 1038 (6.6) | 869 (5.5) |  |

P-values are from chi-square test, all results are N (%)
