# Supplementary material for: Mortality and loss to follow-up among Tuberculosis patients on treatment in Meru County, Kenya: a retrospective cohort study
Source: PLOS Glob Public Health. 2025 Mar 10;5(3):e0003896. doi: 10.1371/journal.pgph.0003896 (PMC11892847; doi:10.1371/journal.pgph.0003896)
Supplement: S2 Table — (DOCX) [file pgph.0003896.s004.docx]

**S2 Table. Participants characteristics stratified by HIV status.**

| **Characteristics** | **HIV status** | | | |
| --- | --- | --- | --- | --- |
|  | **Negative (30186)** | **Positive**  **(6773)** | **Unknown (1061)** | **P-value** |
| Sex |  |  |  |  |
| Male | 22991 (83) | 3794 (14) | 823 (2.9) | <0.001 |
| Female | 7195 (69) | 2979 (29) | 238 (2.3) |  |
| Age in years |  |  |  |  |
| 15 to 24 | 8192 (87) | 766 (8.3) | 275 (2.9) |  |
| 25 to 34 | 9415 (80) | 2108 (18) | 307 (2.6) |  |
| 35 to 44 | 6186 (72) | 2198 (26) | 235 (2.7) |  |
| 45 to 54 | 3112 (71) | 1149 (26) | 122 (2.8) | <0.001 |
| 55 to 64 | 1692 (78) | 430 (20) | 53 (2.4) |  |
| ≥65 | 1589 (89) | 122 (6.9) | 69 (3.9) |  |
| Year of starting TB treatment |  |  |  |  |
| 2012 | 2136 (75) | 633 (22) | 91 (3.2) | <0.001 |
| 2013 | 2410 (77) | 626 (20) | 101 (3.2) |  |
| 2014 | 2626 (79) | 638 (19) | 73 (2.2) |  |
| 2015 | 2472 (82) | 519 (17) | 34 (1.1) |  |
| 2016 | 2372 (81) | 530 (18) | 37 (1.3) |  |
| 2017 | 2992 (78) | 687 (18) | 147 (3.8) |  |
| 2018 | 3481 (82) | 700 (16) | 69 (1.6) |  |
| 2019 | 3142 (81) | 665 (17) | 72 (1.9) |  |
| 2020 | 2775 (82) | 557 (16) | 50 (1.5) |  |
| 2021 | 2769 (77) | 598 (17) | 210 (5.9) |  |
| 2022 | 3011 (79) | 620 (16) | 177 (4.7) |  |
| Treatment facility type |  |  |  |  |
| Public health facility | 24644 (82) | 4451 (15) | 877 (2.9) |  |
| Private health facility | 4892 (67) | 2207 (30) | 163 (2.2) | <0.001 |
| Prisons | 650 (83) | 115 (15) | 21 (2.7) |  |
| BMI group |  |  |  |  |
| Undernourished (BMI<18.5) | 17040 (80) | 3781 (18) | 510 (2.4) | <0.001 |
| Normal (BMI 18.5 to 24.9) | 10017 (80) | 2206 (18) | 350 (2.8) |  |
| Overweight (BMI ≥25) | 1168 (76) | 309 (20) | 52 (3.4) |  |
| Unknown/missing | 1961 (76) | 477 (18) | 149 (5.8) |  |
| TB diagnosis |  |  |  |  |
| Bacteriologically confirmed | 22303 (84) | 3538 (13) | 758 (2.9) | <0.001 |
| Clinical signs and X-ray | 7883 (69) | 3235 (28) | 303 (2.7) |  |
| Patient category |  |  |  |  |
| New case | 1735 (74) | 554 (24) | 44 (1.9) | <0.001 |
| Re-treatment after relapse | 641 (75) | 188 (22) | 22 (2.6) |  |
| Re-treatment after LTFU | 641 (75) | 188 (22) | 22 (2.6) |  |
| Transfer in | 535 (82) | 85 (13) | 32 (4.9) |  |
| Treatment after failure | 185 (90) | 17 (8.3) | 3 (1.5) |  |
| Type of TB |  |  |  |  |
| Pulmonary TB | 26789 (80) | 5688 (17) | 922 (2.8) | <0.001 |
| Extra-Pulmonary TB | 3397 (74) | 1085 (23) | 139 (3.0) |  |
| On recreation drugs |  |  |  |  |
| No | 28917 (79) | 6487 (18) | 1022 (2.8) | 0.69 |
| Yes | 1269 (80) | 286 (18) | 39 (2.5) |  |
| Direct observed treatment (dot) |  |  |  |  |
| Family-based | 28134 (79) | 6303 (18) | 978 (2.8) | 0.62 |
| Community health Volunteer | 208 (79) | 48 (18) | 6 (2.3) |  |
| Healthcare worker | 1844 (79) | 422 (18) | 77 (3.3) |  |
| Treatment regimen |  |  |  |  |
| 2RHZE/4RH | 28701 (80) | 6302 (18) | 1008 (2.8) | <0.001 |
| 2SRHZE/1RHZE/5RHE | 983 (73) | 341 (25) | 26 (1.9) |  |
| 2RHZ/4RH | 242 (80) | 47 (15) | 15 (4.9) |  |
| RHZE/10RH | 143 (73) | 44 (24) | 8 (4.1) |  |
| Others | 117 (73) | 39 (24) | 4 (2.5) |  |
| Nutritional support |  |  |  |  |
| No food support | 5568 (77) | 1436 (20) | 264 (3.6) | <0.001 |
| Therapeutic/Supplementary food | 12281 (82) | 2257 (15) | 386 (2.6) |  |
| Counselling only | 12337 (78) | 3080 (19) | 411 (2.6) |  |
| P-values are from chi-square test, all results are N (%). | | | | |
