# Supplementary material for: Mortality and loss to follow-up among Tuberculosis patients on treatment in Meru County, Kenya: a retrospective cohort study
Source: PLOS Glob Public Health. 2025 Mar 10;5(3):e0003896. doi: 10.1371/journal.pgph.0003896 (PMC11892847; doi:10.1371/journal.pgph.0003896)
Supplement: S3 Table — (DOCX) [file pgph.0003896.s005.docx]

**S3 Table. Univariate analysis of patient characteristics associated with LTFU and deaths within six months of starting TB treatment.**

| **Characteristics** | **LTFU**  **Crude SHR (95%CI)** | | **Mortality**  **Crude SHR (95%CI)** | |
| --- | --- | --- | --- | --- |
|  | CSHR (95%CI) | P-value | CSHR (95%CI) | p-value |
| Sex |  |  |  |  |
| Male | Reference |  | Reference |  |
| Female | 0.64 (0.60‒0.69) | <0.001 | 1.21 (1.14‒1.29) | <0.001 |
| Age in years |  |  |  |  |
| 15 to 24 | Reference |  | Reference |  |
| 25 to 34 | 1.14 (0.99‒1.30) | 0.05 | 1.78 (1.60‒1.99) | <0.001 |
| 35 to 44 | 0.86 (0.75‒0.99) | 0.03 | 2.45 (1.98‒3.02) | <0.001 |
| 45 to 54 | 0.74 (0.60‒0.91) | 0.005 | 3.31 (2.77‒3.97) | <0.001 |
| 55 to 64 | 0.58 (0.42‒0.79) | 0.001 | 4.72 (3.79‒5.88) | <0.001 |
| ≥65 | 0.56 (0.47‒0.66) | <0.001 | 7.43 (5.79‒9.53) | <0.001 |
| Year of starting TB treatment |  |  |  |  |
| 2012 | Reference |  | Reference |  |
| 2013 | 1.03 (0.93‒1.15) | 0.52 | 0.95 (0.74‒1.22) | 0.71 |
| 2014 | 0.74 (0.54‒0.99) | 0.05 | 1.07 (0.81‒1.42) | 0.61 |
| 2015 | 0.65 (0.55‒0.77) | <0.001 | 0.94 (0.71‒1.25) | 0.68 |
| 2016 | 0.81 (0.56‒1.18) | 0.27 | 1.01 (0.76‒1.35) | 0.94 |
| 2017 | 1.04 (0.84‒1.29) | 0.70 | 0.89 (0.70‒1.13) | 0.35 |
| 2018 | 0.76 (0.67‒0.87) | <0.001 | 0.99 (0.69‒1.40) | 0.94 |
| 2019 | 0.71 (0.56‒0.89) | 0.003 | 0.93 (0.71‒1.22) | 0.61 |
| 2020 | 0.63 (0.52‒0.77) | <0.001 | 0.97 (0.76‒1.24) | 0.81 |
| 2021 | 0.79 (0.56‒1.10) | 0.16 | 1.16 (0.96‒1.40) | 0.12 |
| 2022 | 0.58 (0.45‒0.75) | <0.001 | 1.27 (0.99‒1.63) | 0.06 |
| Treatment facility type |  |  |  |  |
| Public health facility | Reference |  | Reference |  |
| Private health facility | 0.71 (0.54‒0.95) | 0.02 | 1.24 (0.95‒1.63) | 0.12 |
| Prisons | 0.70 (0.55‒0.89) | 0.004 | 0.67 (0.45‒0.99) | 0.05 |
| BMI group |  |  |  |  |
| Undernourished (BMI<18.5) | 1.20 (1.06‒1.36) | 0.004 | 1.36 (1.27‒1.46) | <0.001 |
| Normal (BMI 18.5 to 24.9) | Reference |  | Reference |  |
| Overweight (BMI ≥25) | 0.82 (0.64‒1.04) | 0.10 | 1.14 (1.01‒1.28) | 0.04 |
| Unknown/missing | 1.70 (1.35‒2.12) | <0.001 | 2.19 (1.69‒2.84) | <0.001 |
| TB diagnosis |  |  |  |  |
| Bacteriologically confirmed | Reference |  | Reference |  |
| Clinical signs and X-ray | 0.71 (0.62‒0.82) | <0.001 | 2.89 (2.57‒3.25) | <0.001 |
| Patient category |  |  |  |  |
| New case | Reference |  | Reference |  |
| Re-treatment after relapse | 0.87 (0.70‒1.08) | 0.21 | 1.57 (1.37‒1.80) | <0.001 |
| Re-treatment after LTFU | 3.65 (2.89‒4.61) | <0.001 | 1.54 (1.01‒2.37) | 0.04 |
| Transfer in | 1.08 (0.65‒1.80) | 0.77 | 0.71 (0.39‒1.29) | 0.26 |
| Treatment after failure | 2.05 (1.41‒2.99) | <0.001 | 0.98 (0.01‒0.74) | 0.02 |
| Type of TB |  |  |  |  |
| Pulmonary TB | Reference |  | Reference |  |
| Extra-Pulmonary TB | 0.58 (0.47‒0.70) | <0.001 | 1.90 (1.62‒02.22) | <0.001 |
| HIV status |  |  |  |  |
| Negative | Reference |  | Reference |  |
| Infected on ARVs | 1.03 (0.87‒1.21) | 0.76 | 2.86 (2.49‒3.30) | <0.001 |
| Infected not on ARVs | 2.35 (2.00‒2.76) | <0.001 | 5.46 (3.90‒7.64) | <0.001 |
| Unknown/missing | 1.86 (1.32‒2.64) | <0.001 | 2.48 (1.77‒3.46) | <0.001 |
| Other comorbidity | 0.50 (0.27‒0.93) | 0.03 | 4.05 (2.98‒5.48) | <0.001 |
| On recreation drugs | 1.03 (0.85‒1.26) | 0.73 | 1.06 (0.71‒1.58) | 0.78 |
| Direct observed treatment (dot) |  |  |  |  |
| Family-based | Reference |  | Reference |  |
| Community health Volunteer | 1.03 (0.92‒1.15) | 0.58 | 1.06 (0.65‒1.72) | 0.83 |
| Healthcare worker | 1.08 (0.43‒2.68) | 0.88 | 1.11 (0.79‒1.56) | 0.55 |
| Treatment regimen |  |  |  |  |
| 2RHZE/4RH | Reference |  | Reference |  |
| 2SRHZE/1RHZE/5RHE | 2.03 (1.53‒2.68) | <0.001 | 1.53 (1.36‒1.72) | <0.001 |
| 2RHZ/4RH | 0.64 (0.33‒1.24) | 0.19 | 0.85 (0.45‒1.64) | 0.64 |
| RHZE/10RH | 1.17 (0.62‒2.21) | 0.63 | 2.14 (1.67‒2.73) | <0.001 |
| Others | 0.09 (0.04‒0.25) | <0.001 | 0.74 (0.21‒2.53) | 0.63 |

CSHR; Crude Sub-distribution Hazard Ratio, CSHR are from multilevel competing risk analysis models.
