# Supplementary material for: Mortality and loss to follow-up among Tuberculosis patients on treatment in Meru County, Kenya: a retrospective cohort study
Source: PLOS Glob Public Health. 2025 Mar 10;5(3):e0003896. doi: 10.1371/journal.pgph.0003896 (PMC11892847; doi:10.1371/journal.pgph.0003896)
Supplement: Attachment — Submitted filename: Response to Reviewers.docx [file pgph.0003896.s006.docx]

## Journal Requirements:

1. Please provide an Author Summary. This should appear in your manuscript between the Abstract (if applicable) and the Introduction, and should be 150–200 words long. The aim should be to make your findings accessible to a wide audience that includes both scientists and non-scientists.

We have added the author summary after the abstract (lines 56/67).

2. We have noticed that you have uploaded Supporting Information files, but you have not included a list of legends. Please add a full list of legends for your Supporting Information files after the references list.

We have added the supporting information captions after the references list (Lines 655/661).

## Reviewers' comments:

### Reviewer #1:

This manuscript examines loss to follow up and mortality rates for patients with TB in Meru country over a 10-year period, 2012-2022. The authors highlight the high rate of loss to follow-up as well as the high mortality rates facing PLHIV, particularly during the early phase of TB treatment. Despite some ambiguities, the manuscript is well-written and the statistical methods are sound.

We thank the reviewer for these comments which have greatly improved the manuscript.

I provide below specific comments for the authors to address in the manuscript:
Title
• Line 1: Please correct “lost” to “loss.”

The word lost has been replaced with loss and the change has been made in other relevant sections

Abstract
• Line 26: Please edit “lost-to-follow-up” to “lost to follow-up.”

The words “lost-to-follow-up” has been replaced with “lost to follow-up.” Line 26.

• Line 42. The sentence starting on line 42 is ambiguous. I suggest revising it to emphasize the high mortality among HIV/TB co-infected persons not on ARV.

The sentence has been rephased to read “HIV infected patients on ARVs, not on ARVs and unknown HIV status versus not infected were positively associated with mortality.” Lines 42/44.

• Line 46. Last sentence of the abstract. The sentence starting on line 46: “Targeted interventions are needed to improve treatment outcomes for TB patients who are at high risk of death or being LTFU.”

Thank you for this suggestion. We have replaced the sentence with the suggested wordings (Lines 46/47).

Introduction
• Line 63. Edit “bare” to “bear.”

This has been corrected now (line 79).

• Line 69. Edit “Lost” to “Loss.”

We have corrected this (line 88).

• Line 83: I suggest editing “studied” to “examined” to avoid repetition.
Thanks for the suggestion, we have updated the sentence (line 102).

Materials and Methods
• Line 99: Please spell out “Mt” to “Mount.”

The word mount has been written if full (line 118).

• Line 131: Please edit “captures and stores” to “capture and store”

This has been corrected (line 155)

• Line 149: I suggest editing the sentence starting on this line to the following: “Loss to follow-up was defined to include TB patients who initiated TB treatment but experienced an interruption lasting at least two consecutive months. Treatment success was defined as those who completed…”

Thanks for the comment. We have updated the sentences to reflect the suggestion (lines 175/176).

• Line 154: Please insert the word “sample” between study and size.

The word sample has been inserted (line 182).

• Line 155: I suggest removing “presumptive” since the patients included in the study had received a confirmation of TB diagnosis either bacteriologically or clinically with chest X-ray findings consistent with TB.

The word presumptive has been removed from the sentence. We have also added the words “drug-susceptible” to reflect the study population. As explained elsewhere, only drug-susceptible TB patients were included in study (line 181).

• Line 157: Please edit “clinical” to “clinically”

The word clinical has been replaced with clinically (line 183).

Results
• Line 209: clarify ”more frequently” by stating the actual percentage of those who received care in a public health facility.

We have made this clearer by adding the n and % (line 235).

• Line 210: I suggest stating the percentage of those were underweight instead of overweight.

We concur with the reviewer and have added the proportion of underweight patients (lines 236/237).

• Line 213-214: Correct “GenXpert” to “GeneXpert.”

This has been corrected in the whole manuscript (Lines 240, 241).

• Line 224: Correct “Thousands” to “thousand” and “months” to “month.”

We have made the correction (line 250).

• Line 230: Correct “extra-Pulmonary” to “extra-pulmonary.”
We have replaced the word extra-Pulmonary with extra-pulmonary (line 256) and other relevant sections throughout the manuscript.

• Line 236: Please include “%” after “5.1”

We have added % to read 5.1% (line 263).

• Line 238: Please edit “resistant” to “drug-resistant TB.”
We have replaced the word resistant with drug-resistant TB (line 264).

Discussion
• Line 301: Please correct “co-effected” to “co-infected.”

This has been corrected (line 328)

• Line 329: You should also consider the possibility of emerging drug resistance in patients receiving treatment after relapse as an explanation for the high mortality in patients on TB treatment after experiencing relapse. This would argue for drug susceptibility testing and appropriate treatment regimens for such patients.

Thank you for this suggestion. We have added a sentence suggesting the relapse patients might have higher risk of TB drug resistance (lines358/359) and recommended systematic drug susceptibility testing for this population (lines 404/405).

• Line 343: The word “threat” is considered stigmatizing language in describing persons with TB. I suggest replacing it with “challenge.”

We concur with the review. We have replaced the word threat with challenge as suggested (line 373).

• Line 358: Please edit “new” to “newly.”

We have corrected this (line 388)

• Line 372: Please edit “resources contrains” to “resource constraints.”

We have corrected the spelling mistake (line 402).

• Line 374: I suggest rephrasing the last sentence to state: “A multi-sectoral approach is thus necessary for successful TB treatment.” or “A multi-sectoral approach is thus necessary to improve TB treatment outcomes.”

We have rephrased the sentence to read “A multi-sectoral approach is thus necessary to improve TB treatment outcomes” (line 405/406).

Tables:
• Table 1: Please edit the “$” sign from “On recreation drugs$”

This has been edited to a superscript to show that is a symbol. The use of the symbol is explained in the table footnote (page 30, Table 1).

• Table 1: Capitalize “dot” to “DOT”

The word “dot” has been replaced with “DOT” (page 30, Table 1).

• Table 2, Line 637: Please edit “Lost-to-follow-up” to “Loss to follow-up.”
This has been corrected (Page 31, line 679).

Other comments
Please clarify whether drug resistance was available in Meru County. Do persons with drug-resistant TB have access to appropriate treatment in Meru County or do they have to travel to other locations?

Meru County like any other county in Kenya, maintains two separate databases for drug-susceptible and drug-resistant TB patients. This study only used drug-susceptible TB patients. The drug-resistant TB patients are managed using standardized regimen following WHO 2018 guidelines. We have explained this in lines 139/142 and specified that the study population were only drug-susceptible TB patients.

It is unclear whether reasons for TB treatment interruptions are documented in the TIBU electronic system. Can the authors clarify whether some of the treatment interruptions could have been due to adverse drug events?

Unfortunately, the TIBU electronic system doesn’t record the reasons for TB treatment interruptions because this is passive surveillance i.e the health workers don’t actively trace patients who miss their monthly clinic visits. We have added this to the study limitation (Lines 415/416).

### Reviewer #2:

 This is a well- written paper with appropriate analytical questions for LTFU and death to describe the TB response in Meru County, Kenya. The study addresses LTFU and mortality rates which are rarely studied.

Thank you for the complement.

Line 66: what is the TB case and mortality rate for Kenya for comparison to Meru findings

We have added the latest statistics from Kenya national TB program (2022) on mortality and loss to follow-up among TB cases in Kenya. Our findings are within the Kenya national statistics reported (line 83/86).

Line 246: Mortality rate instead of incidence rate?

Thanks for highlighting this, we have made the correction (line 274).

Line234: is this a low cure rate?

In 2022, the treatment success rate in Kenya was 86%. Therefore the 85% observed in this study was within the Kenya national range. The cure rate of 54% (among those bacteriologically confirmed TB cases) reported in our study is lower than the 76% national figure. This is likely because sputum smear test was not systematically performed at month 5 and 6 of treatment in this setting.

Line 232: Was there a trend in re-treatment of LTFU to describe program efforts to track TB patients? What proportion of LTFU were not re-treated.

We agree this is an important aspect of tracking program performance, however, there was no linear trend in annual re-treatment of LTFU patients (trend P-value=0.39). We don’t have data on proportion of LTFU not re-treated. From an ongoing study (not published), we were able to only trace and restart treatment among 35% of those LTFU.

Line 277: Did an age/sex analysis show anything interesting? HIV analyses generally are conducted at this level for all clinical cascade elements to target program efforts.
We did not find any significant differences in sex apart from lower risk of LTFU among the female. However, we found significant differences by age including a linear trend in risk of mortality (table 3 & Figure 2b). We have a supplementary Table (S1 Table) of all the patient characteristics stratified by age.

Titles would be helpful for graphs.

Graph titles are provided in the figure legend (line 662/669).

What went right in Mery Central Sub-County?

Meru Central Sub-County had received some support to actively follow-up the patients from starting treatment up to the end. This involved community health promoters escorting patient to clinic to pick their drugs every month. This was expensive, the support stopped during COVID-19 pandemic. We have added a sentence in the discussion to explain this (lines 368/370).
